# Timely poacher detection and localization using sentinel animal movement

**DOI:** 10.1038/s41598-021-83800-1

**Published:** 2021-02-25

**Authors:** Henrik J. de Knegt, Jasper A. J. Eikelboom, Frank van Langevelde, W. François Spruyt, Herbert H. T. Prins

**Affiliations:** 1 Wildlife Ecology and Conservation Group, Wageningen University and Research, Droevendaalsesteeg 3a, 6708 PB Wageningen, The Netherlands; 2 School of Life Sciences, University of KwaZulu‐Natal, Westville Campus, Durban, 4000 South Africa; 3 Welgevonden Game Reserve, P.O. Box 433, Vaalwater, South Africa; 4 Department of Animal Sciences, Wageningen University and Research, De Elst 1, 6708 WD Wageningen, The Netherlands

**Keywords:** Conservation biology, Machine learning

## Abstract

Wildlife crime is one of the most profitable illegal industries worldwide. Current actions to reduce it are far from effective and fail to prevent population declines of many endangered species, pressing the need for innovative anti-poaching solutions. Here, we propose and test a poacher early warning system that is based on the movement responses of non-targeted sentinel animals, which naturally respond to threats by fleeing and changing herd topology. We analyzed human-evasive movement patterns of 135 mammalian savanna herbivores of four different species, using an internet-of-things architecture with wearable sensors, wireless data transmission and machine learning algorithms. We show that the presence of human intruders can be accurately detected (86.1% accuracy) and localized (less than 500 m error in 54.2% of the experimentally staged intrusions) by algorithmically identifying characteristic changes in sentinel movement. These behavioral signatures include, among others, an increase in movement speed, energy expenditure, body acceleration, directional persistence and herd coherence, and a decrease in suitability of selected habitat. The key to successful identification of these signatures lies in identifying systematic deviations from normal behavior under similar conditions, such as season, time of day and habitat. We also show that the indirect costs of predation are not limited to vigilance, but also include (1) long, high-speed flights; (2) energetically costly flight paths; and (3) suboptimal habitat selection during flights. The combination of wireless biologging, predictive analytics and sentinel animal behavior can benefit wildlife conservation via early poacher detection, but also solve challenges related to surveillance, safety and health.

## Introduction

Wildlife trade is a low-risk, yet high-profit crime, ranking fourth in terms of revenue after trade in drugs, humans and arms^1^. Wildlife crime is driven by a rapidly expanding wealthy class in some cultures that views animal parts as medicine or status-enhancing luxury goods^2^. The demand for animal parts has led to escalating prices^3^, which consequently fuels poaching. As one of the main causes for biodiversity decline^4^, poaching increasingly threatens the existence of wildlife, notably pangolins, rhinos, elephants and tigers. Ultimately, losses of these and other species can reshape entire ecosystems via cascading effects.

Although the ultimate solution is to reduce the global demand for wildlife products, efforts to do so have not been successful enough^5^. Local efforts thus often aim at deterring poachers, mainly through ranger patrols. Deadly force used by poachers incites conservation authorities into intensified ‘militarized conservation’, resulting in frequent shootouts between poachers and conservation officers^6^. Sadly, poaching of wildlife still continues to be a threat to the preservation of many wildlife species^1^, as anti-poaching rangers often arrive too late at crime scenes^7^. An effective method for early poacher detection and localization is thus urgently needed, so that preventive action can be taken. With situational awareness, law enforcers can operate under safer conditions with reduced risk of fatalities and potential to de-escalate conflicts. An effective poacher early warning system (EWS) thus contributes to preventing lethal violence, not only against wildlife, but also against conservation officers and poachers^6^.

Animal sentinels, especially those that are abundant and no targets themselves, may provide an early warning that poachers are en route. Prey species may be good sentinels as these species have evolved a suite of traits aimed at preventing them from being killed, e.g., via early predator detection and escape^8^. This often extrapolates to humans as well, since many prey species evolved together with human hunters, leading to anthropogenic disturbance stimuli triggering similar, or often even stronger, evasive responses^9,10^. Until now, practical constraints have hampered the development of a sentinel-based EWS^11^. Although wireless sensors can generate large volumes of data, the areas in which poaching occurs often lack infrastructure that allows real-time wireless communication of sufficient bandwidth^7^. Moreover, animal behavior is known to be complex and context-dependent, thus an EWS needs to be able to handle rich contextual data when identifying behavioral abnormalities linked to anthropogenic disturbances. Fortunately, advances in technology, computing and analytics have now alleviated these constraints^12^. We therefore tested the concept of whether the behavior of sentinel animals can be used to detect and localize human intrusions using wearable biologging sensors and predictive algorithms (Supplementary Fig. S1).

**Figure 1:**
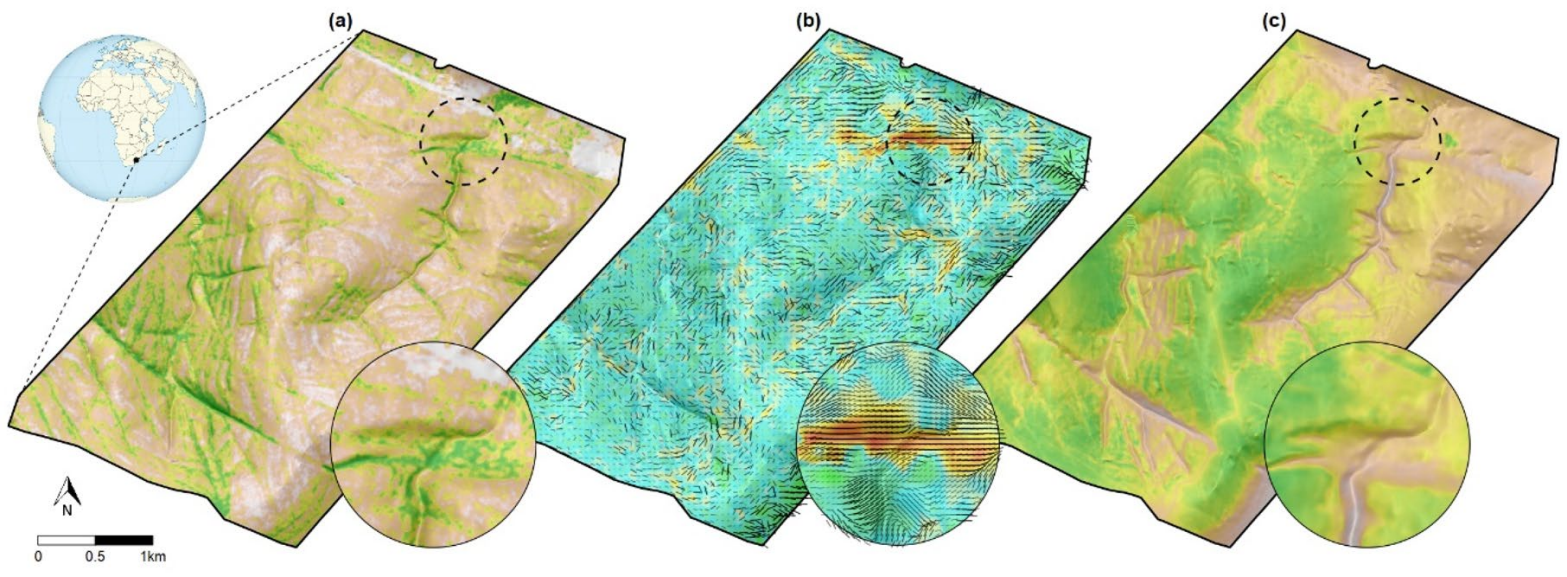
Overview of the study area with three examples of how normal behavior varies spatially: (**a**) topography and tree cover in the study area (white to green with increasing tree cover); (**b**) movement speed (third quartile) and directionality of wildebeest during the afternoon (blue to red with increasing speed; length and darkness of line segments indicates the degree of directional preference and orientation indicates the preferred movement direction); and (**c**) modelled habitat suitability of wildebeest during the afternoon as function of habitat characteristics (white to green with increasing suitability). The inset figures exemplify the importance of considering environmental context in the early warning system, since fast, straight and directional movements through low suitability areas are part of the sentinels’ normal behavior. Thus, solely detecting fast and straight movements may not suffice as early warning indicators. All maps were generated in R3.5.0 using GIS, location and modelled data^49^.

We tested the sentinel-based EWS in an African savanna, home to several targeted species (e.g., pangolin, elephant, rhino and lion) that coexist with an assemblage of mammalian prey species that could be potential sentinels. We deployed wearable GPS and tri-axial accelerometer sensors on 138 animals equally over four species (plains zebra, blue wildebeest, common eland and impala) in a 1200 ha fenced, predator-free area inside Welgevonden Game Reserve (WGR), South Africa (Fig. 1). These sensors transmitted data wirelessly via a LoRa network connected to a backhaul. During a period of 7 months, WGR park officials executed 57 intrusions mimicking poachers (referred to as ‘experimental intrusions’). Data collected in the absence of experimental intrusions were used to characterize undisturbed behavior, allowing quantification of the degree of abnormality of movement behavior at any point in time. During all these experimental intrusions and matched controls, a median of 47 sensors yielded data for further analyses.

We engineered a large set of potentially meaningful and ecologically relevant features, describing the geometry of individual trajectories as well as emergent herd topologies and various characteristics of the animal-environment interplay (split into 4 main classes: individual geometry, accelerometer-based, collective movement features, and indices of space usage; and 12 sub-classes; and various standardizations of features to capture deviations from normal behavior, see Supplementary Table S1). Then, we applied a multi-step dimensionality reduction approach (first across a subset of features within sub-classes to collapse the ecologically related features into a low-dimensional characterization, then across the set of selected principal components from all classes to reduce multicollinearity; see “Methods” section) and segmented the dataset into experimental intrusions and controls. Data during experimental intrusions were randomly matched with control data of the same period, one or 2 days earlier or later, when no intrusion took place. To generate predictive signatures for the EWS, we followed a three-step process: (1) behavioral response classification focusing on detecting evasive anti-predator behavior by each individual separately, followed by (2) intrusion detection focusing on a system classification through integrating signals over all individuals, and (3) intrusion localization. We allocated each experimental intrusion or control segment to either the training phase or the evaluation phase, applying a leave-one-group-out cross-validation approach on these segments to make the best use of all data (see “Methods” section for details).

## Results

**Figure 2:**
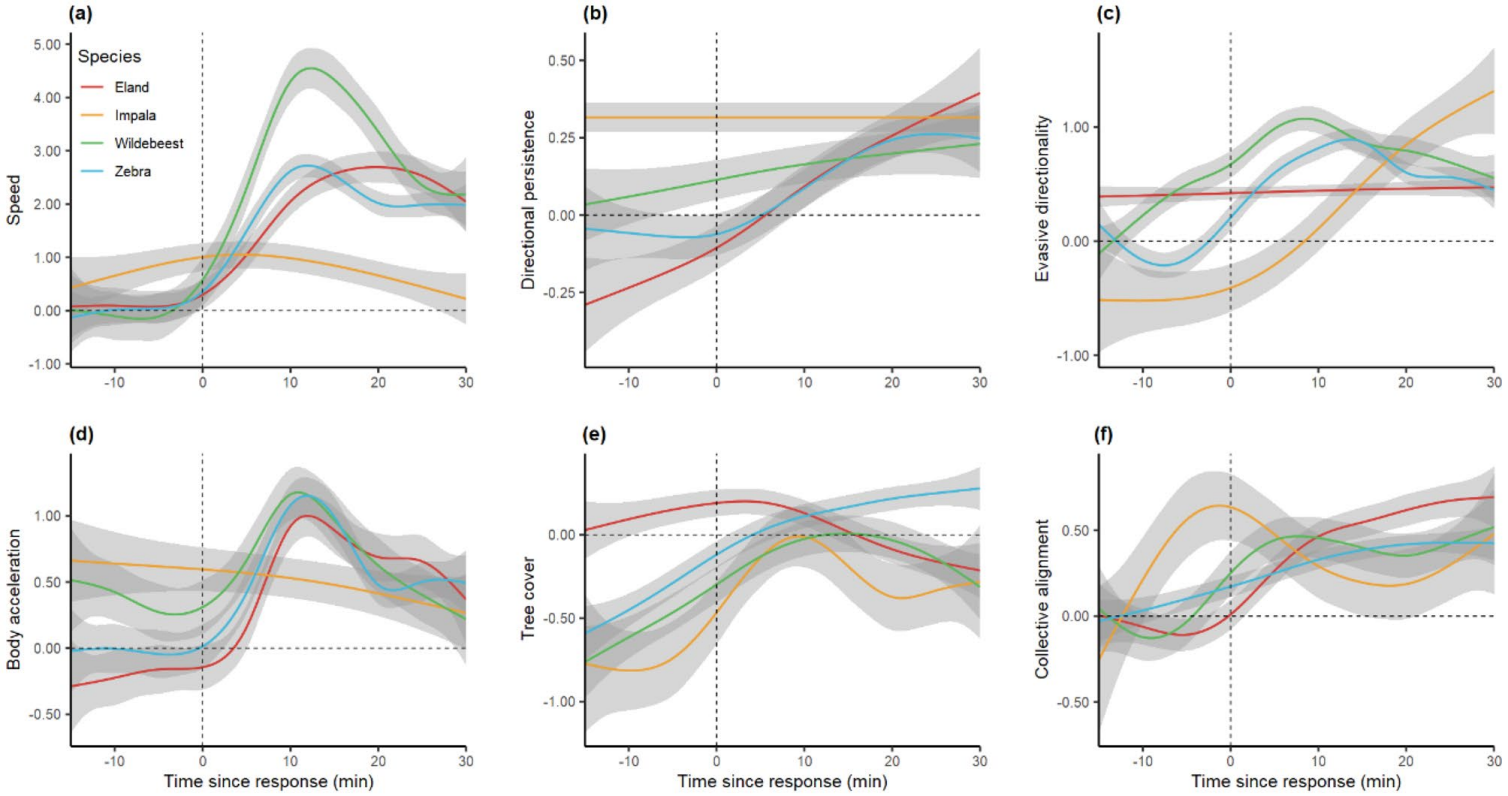
A sample of the 2117 computed animal movement features characterizing the sentinels’ behavior near experimental intrusions, shown here as function of the time since the annotated start of their response behavior (i.e., ‘flight’ and ‘regroup’ as described in the main text). All y-axes show standardized values (zero-mean and unit-variance when undisturbed), and the shaded area around each line (i.e., sentinel species) depicts pointwise 95% CI of a General Additive Model. When encountering the experimental intrusions, the sentinels moved faster (**a**), straighter (**b**), away from the intrusion (**c**), and with higher body acceleration (**d**). The sentinel species that prefer more grass-dominated habitats (i.e., lower tree cover) tended to move towards areas with higher tree cover (**e**) and thus lower habitat suitability. Moreover, encountering the intrusions induced more aligned collective movement (**f**).

**Figure 3:**
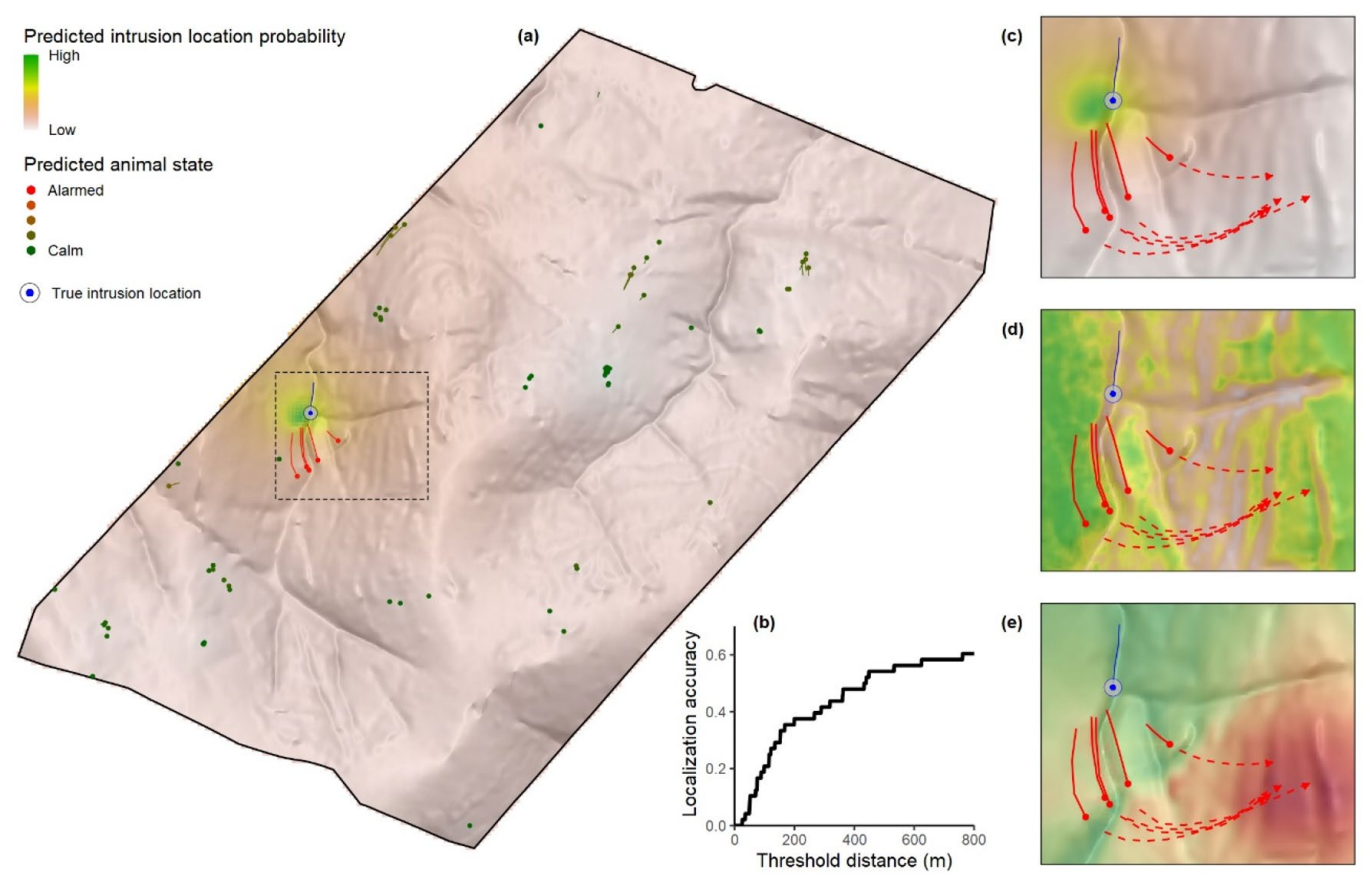
Spatial performance of our early warning system. (**a**) The predicted spatial probability surface for the intrusion’s location (based on data from the sentinel animals only) for one of the experimental intrusions. For all experiments where the intrusion was algorithmically detected (82.5%), the spatial localization accuracy as function of threshold distance (**b**) that 54.2% of these correctly detected intrusions could be localized with a spatial error of less than 500 m and 20.8% within 100 m. The dashed focal area shown in (**a**) is highlighted in (**c**–**e**), where the sentinels’ (here: wildebeest) movements in the next 10 min is indicated with dashed lines. (**c**) The spatial localization prediction of the intrusion. The evasive movements of the fleeing wildebeest are fast compared to their normal movement at that location (Fig. 1b), and highly aligned. While fleeing, the wildebeest move through habitat with a low suitability (**d**, see Fig. 1c), and towards areas that are energetically costly to reach (**e**, movement costs are computed based on topography and relative to their current position, where the cost of movement is assumed to be inversely proportional to movement speed on an incline as computed using Tobler's hiking function). The experimental intrusion as depicted in this figure is animated in Supplementary Movie 1, including output from the animal classification and intrusion localization algorithms. All maps were generated in R3.5.0 using GIS, location and modelled data^49^.

Exploration of the animals’ reaction to the experimental intrusions highlighted several broad characterizations of their response. First, the experimental intrusions triggered nearby sentinels to divert their movement away from the perceived treat while increasing their speed, body acceleration and directional persistence (Fig. 2). This, together with elevated variation in such features, resulted in more directional, brisk, straight and erratic movements. These evasive flights lasted on average 47 min per fleeing group of zebra (SD = 28, n = 29), 39 min for wildebeest (SD = 33, n = 15), 46 min for eland (SD = 18, n = 15), and 43 min for impala (SD = 14, n = 14). Second, the difference between the sentinels’ response behavior and their normal behavior was larger when comparing the individuals’ movement in the same spatial (location and habitat) and temporal (seasonal and diurnal) context. Third, the sentinels selected sub-optimal habitat and chose flight paths that incurred higher energetic costs via faster and uncommon uphill movement in response to the experimental intrusions, possibly in an effort to find refuge (Figs. 2, 3). Fourth, apart from alterations in the geometry of individual movement trajectories, patterns of collective geometry changed in the vicinity of the experimental intrusions. Generally, nearby individuals tended to form groups with more synchronized and aligned movements (Fig. 2f).

We trained a support vector machine (SVM) to algorithmically classify the animal’s response behavior as either undisturbed (i.e., calm or normal) or disturbed (a summary label for the above-described responses). We were able to achieve an average precision of classification (i.e., the area under the precision-recall curve) of 46%. Depending on the chosen value of the response probability decision boundary, the classification performance achieved up to 100% precision, or 100% recall, with a maximum *F*_1_-score of 47% (Supplementary Fig. S2). Comparing the SVM’s average precision on various subsets of the data resulted in three noteworthy variations in predictability of response behavior: (1) intrusion type (on foot 52%, by vehicle 14%), (2) species (eland 47%, impala 17%, wildebeest 29%, zebra 57%), and (3) time of day (morning 48%, noon 26%, afternoon 53%). A higher predictability near humans on foot compared to motorized vehicles suggests a stronger behavioral response to the former and is in line with other findings^8,13^. A lower predictability for impala and wildebeest may imply that these animals exhibit a broader suite of response behaviors, possibly including antipredator responses not included here (e.g., ‘freezing’ or ‘threat inspection’ behavior). For impala, being the smallest of our sentinel species, it may furthermore be caused by a high-quality food requirement inducing them to delay escape and hence reduce the associated opportunity costs^8^. The lower predictability around noon could be due to the midday heat inducing animals to accept a higher risk and lower their energy expenditure of costly risk-avoidance behavior, thereby creating less pronounced signatures in the data.

Following animal behavior classification, we were able to distinguish intrusions from controls with 86.1% accuracy (82.6% precision, 89.2% recall) using logistic regression, exclusively using the movement data of the sentinels (Table 1). The odds of an intrusion increased considerably with higher SVM-predicted probabilities of response behavior, the degree of local spatial autocorrelation therein, and a decrease in spatial clustering of sentinels that were predicted to be undisturbed. Including more features in the detection classifier boosted its predictive accuracy to 91% (Supplementary Fig. S4), but also increased the risk of lowering its generalizability to other areas due to potential overfitting. The true positive rate was 84.2%, and there was no apparent positive relationship between the probability that an intrusion was correctly detected and the number of working sensors (logistic regression, *p* = 0.260).

**Table 1:** Confusion matrix of the poacher detection algorithm. Bold numbers indicate correct predictions.

**Prediction**	Truth	
	Control	Intrusion
Control	**52**	10
Intrusion	5	**47**

Following detection, we predicted the location of the intrusion relative to the position, movement direction and SVM-predicted response probabilities of the sentinels. We summarized the performance of the localization prediction through the Euclidian distance between the peak prediction and the true location of the intrusion, followed by computing the spatial error of the 10 most dense probability surfaces per experimental intrusion. In 20.8% of them the predictions were highly accurate, namely within 100 m from the true location, increasing to 41.7% and 54.2% respectively, for distances up 300 m and 500 m (Fig. 3b).

## Discussion

Our study thus clearly demonstrates that sentinel animal behavior can be used to detect poachers, since predictable signatures in behavioral responses to disturbance stimuli can be used to detect and locate human intrusions. Indeed, the sentinels took systematic and detectable evasive action when experimental intruders came near.

The sentinels increased their movement speed and body acceleration as they generally do during anti-predator responses^8^, whilst moving away from the perceived threat with higher directional persistence (Fig. 2). They did so for a considerable amount of time per flight response (45 min on average), longer than only instantaneously running away, thereby substantially trading off energy for safety^14^. This signal became even more pronounced in the context of the individuals’ normal behavior given the prevailing conditions (season, time of day and habitat), since a systematic deviation from normality is key to successful identification of disturbed behavior. It thus proved to be important to explicitly consider the spatial–temporal context of the movement-environment interplay when using sentinel movement metrics as early warning indicators. Solely using movement speed as indicator^15^ without incorporating environmental conditions is therefore not very informative (Fig. 1).

These findings suggest that the sentinels elevated their energy expenditure while fleeing, in line with theory on energy landscapes and the landscape of fear^8,14,16,17^. However, not only did experimental intrusions trigger faster-than-normal movement, but the sentinels also tended to utilize the terrain by moving uphill, thereby increasing their energy expenditure (Fig. 3). Moreover, the sentinels seemed to alter their decision-making during evasive actions, selecting less optimal habitat than they would do when undisturbed (Figs. 2e, 3d). This suggests that anti-predator trade-offs relate to energy trade-offs and that perceived threats can induce resource avoidance^18^. Together, these consequences of anti-predator behavior can incur significant energetic and opportunity costs^18^. These energetic costs are generally not considered in the indirect costs of predation within the landscape of fear framework, but are now increasingly being recognized^14,18^. Our findings suggest that anti-predator behavior not only incur costs in terms of trading off foraging and resting for vigilance, but also in terms of increased costs due to (1) performing long, high-speed flights; (2) choosing energetically costly flight paths; and (3) selecting suboptimal habitats during flights.

Although the study of collective behavior of animals within groups has predominantly relied on controlled laboratory-based studies and theoretical models^19,20^, our high-resolution data on manifold large terrestrial mammals allowed the detailed computation of collective movement properties in their natural habitat in relation to perceived threats. The sentinels increased group coherence when intruders were near (Fig. 2f), presumably in an effort to find safety in numbers^21^, whilst at the same time avoiding the likelihood of collisions by increasing alignment during escape^22^. These findings support predictions from theoretical studies^23^ and controlled laboratory experiments^24^.

Central to these findings is that the responsive and evasive behavior of animal sentinels can be used to algorithmically detect and localize poachers. A sentinel-based EWS is robust against adaptive behavior of poachers, as an abundance of sentinels cannot easily be manipulated and fooled^25,26^. Additionally, shooting sentinel animals would give away the poacher’s position, both via its acoustic signal^7^ as well as through the sensor data of the shot animal. Moreover, if hackers were to tap into the dataflow, only the locations of the sentinels may be revealed, but not those of targeted species. Applying biologging technology directly to targeted species is risky, and will rule out preventive intervention as it only enables the post hoc identification of mortalities^7^. Instead, the responsive behavior of untargeted sentinels crossing path with poachers en route provides an early warning and situational awareness to anti-poaching personnel.

Our proposed sentinel-based EWS critically hinges on the premise that sentinel animals respond reactively to human intrusions. This requires that these sentinels have evolved with natural and human predators, and that they have maintained their anti-predator behavior^27^. In African savannas, apex predators like lion and leopard are generally present and fear of the human “super-predator”^28,29^ is pervasive throughout mammal communities in Africa^30^. However, empirical evidence shows that response to natural predators and humans varies across contexts and with predator mode: sit-and-wait ambush predators induce different responses than cursorial predators^31,32^, and humans on foot are generally more evocative than other anthropogenic stimuli (e.g., motorized vehicles)^8,13^. To prey, illicit human activity in conservation areas may be rarer and less predictable than encounters with natural predators. Human encounters could therefore be more stressful, since lack of predictability is a well-established trigger of reactive responses like flight^33^. Several studies suggest that free-roaming animals not only respond differently to human presence than to natural predators, but also that human presence evokes stronger responses^9,34,35^. Since our study was intentionally conducted in a predator-free environment, the next logical step is to include the sentinels’ responses to their natural predators in the EWS. Although we currently lack the knowledge and data to separate human-induced from predator-induced behavioral shifts in wild-living animals^36,37^, the behavior and approach movements of natural predators is expected to be sufficiently different from that of humans to successfully do so.

The main advantage of our proposed sentinel-based EWS is its ability to filter out periods without poaching activity, thereby prioritizing model sensitivity over specificity. However, in African savannas it is generally a rare event for a sentinel to encounter a human. Therefore, given our current false positive rate of 8.8%, many false positives will be generated over time when an EWS is actually deployed. The poacher detections by the EWS will thus require an extra layer of verification by, e.g., visually inspecting the patterns in localizations generated by the EWS or dispatching an Unmanned Aerial Vehicle to the detected poacher location. Known locations of legal human activity should then be taken into account as well, e.g., roads or camps with tourists who could trigger responses by animals. The role of this EWS is not that of a fully automatic system to directly dispatch an anti-poaching unit, but to help wildlife reserves make informed decisions about managing their anti-poaching resources.

Using animal sentinels as a lens to the environment is in itself not new, as they have long been employed to detect human exposure to biological and chemical hazards (e.g., canaries in coal mines)^38,39^, and more recently to detect the onset of natural disasters^40,41^, epileptic seizures^42^ or outbursts of violence^43^. Elucidating the hitherto hidden information in the behavior of animals with cutting-edge technology can help us gauge the conditions of life on Earth^44^. More specifically, this approach can expose illicit human activities, such as illegal fishing^45^ and, as shown here, poaching. Our study is the first to document the use of untargeted sentinel behavior as an early warning against wildlife crime, yet our approach is generalizable beyond animals as sentinels. Similar methods could be utilized to detect anomalous behavior of people in crowds in response to a perceived threat^46^. Harnessing the collective sensing capacities of sentinels will thus not only innovate wildlife conservation and help turn protected areas into safe havens, it has the potential to advance many other applications as well.

## Methods

### Study system and species

This study was performed in Welgevonden Game Reserve (WGR), a privately owned game reserve in the Limpopo province, South Africa (24° 10′ S; 27° 45′ E to 24° 25′ S; 27° 56′ E). The reserve is located in the mountainous Waterberg region. WGR was established on former agricultural lands in the early 1980s and the main occurring vegetation types are Waterberg Mountain Bushveld and Sour Bushveld. The Waterberg region has a temperate climate, with two distinct seasons, characterized by the rainfall regime: a dry season ranging from April to September and a wet season ranging from October to March, with an average annual precipitation in WGR of 634 mm. Our study area is an enclosed breeding camp within WGR, with a size of approximately 1200 ha. Main predator species such as lion, cheetah and spotted hyena were excluded from this study area, as well as elephant and rhino.

WGR equipped 35 impala (*Aepyceros melampus*), 34 blue wildebeest (*Connochaetes taurinus*), 35 plains zebra (*Equus burchellii*) and 34 common eland (*Taurotragus oryx*) with a GPS and accelerometer sensor equipped custom made collar; an estimated 23% of the individual impalas present in the area, 48% of the eland, 40% of the wildebeest and 40% of the zebra. However, due to malfunctioning and errors made in the sensor development process, only 83 of the sensors yielded data at any point in time, thus lowering the effective density of sentinel animals. During the experimental intrusions (see below), the median number of data-yielding sensors was 47, and minimally 30. The animal movement data were recorded day and night and transmitted wirelessly in near real-time to five long-range low-power LoRa radiocommunication gateways in the study area, from where data packages were routed to an on-line data warehouse via a 3G/4G backhaul. The deployment of these sentinel animals were approved by the board and CEO of WGR as a management action and was performed in accordance with relevant guidelines and regulations (see Supplementary GPS Collaring letter).

### Experimental intrusions

Between September 2017 and March 2018, WGR employees performed experimental intrusions (lasting ca. 2 h) on foot and by car through the study area, at varying locations and movement routes through the study area, independent from the locations of the sentinel animals. The movement of the intrusions were tracked by GPS, and the relevant metadata for each intrusion recorded (mode of transport, group size, start time, end time). The intrusions were distributed in a stratified way over the mornings, middays and afternoons (with time slots relative to specific solar positions: sunrise, solar noon and sunset). Furthermore, the intrusions were temporally spread in such a way to avoid a disturbance overflow for the sentinel animals, by performing a maximum of five experiments per week and a maximum of two experiments per day (and then only with one intrusion in the morning and one in the afternoon).

### Data gathering

The animal sensors gathered location data via GPS and overall dynamic body accelerations^47^ (ODBA) via a tri-axial accelerometer (range ± 2 g; sampling frequency 100 Hz, down-sampled to 10 Hz prior to analysis). The GPS was scheduled to record spatial position at irregular intervals depending on the level of activity as gauged by ODBA. All sensors were scheduled to record locations every 15 min in the absence of sufficient activity (given that successive fixes were further than 5 m apart, else a geofence was applied and the new coordinate was omitted to save bandwidth and battery power, thereby assuming that the animal still was at its previous location). The GPS fix rate was increased up to 2- or 10-min intervals (depending on two different sensor settings) when ODBA indicated sufficient activity (after checking for the geofence). ODBA data were sampled continuously and summarized per 15 s window in a mean, maximum and variance value.

The experimentally intruding groups were outfitted with handheld GPS devices that recorded their location every 5 s and these groups logged and timestamped all their pre-defined activities and metadata on a tablet using CyberTracker^48^ during their intrusion. Most cars traveling through the study area were tracked by GPS as well to filter the animal data for disturbances by cars unrelated to the experimental intrusions.

Weather data (temperature, radiation, precipitation and wind) in the study area were recorded on a 3-min resolution with a weather station in the north of the study area. We assumed the 1200 ha study area to be sufficiently small to assume the weather station data to be representable for the prevailing weather conditions throughout the study area. GIS data of the study area (summarized in Supplementary Table S1) consisted of information on topography, infrastructure (e.g., fences, roads, powerlines, etc.) and vegetation cover (supervised classification of 25 cm resolution aerial imagery into four classes: trees, herbaceous/grass, sand/soil and other/built-up area).

All further data processing and algorithm development was done in the software R3.5.0^49^.

### Data pre-processing

To link the animal location data with the intrusion location data, as well as to correct for the substantial level of positional noise present in the animal location data, we modelled the animal location data to regular 1-min resolution trajectories using the following five steps. First, we filtered out large obvious errors (e.g., obvious outliers and irregularities such as locations far outside the study area) from the data. Second, we corrected systematic medium-scale outliers: ‘spikes’ that occurred due to positional outliers. Such spike-like outliers were visible during sensor testing while following known straight-line trajectories along an airstrip, thereby confirming that these spike-like geometries most likely resulted from positional error rather than true animal movement. Points were classified as anomalous spike points when (a) the displacement to and away from this point was high (> 500 m), (b) when the distance between the locations before and after this point was small, and (c) when the turning angle at this point approached 180 degrees. Therefore, we corrected the locations that were classified as spike-like anomalies by shifting them closer to the straight line between the neighboring points. The extent of this shift was set relative to the degree of spikiness of the points (the spikier the pattern, the larger the shift towards the midpoint of the adjacent coordinates). Third, after filtering and correcting the original locations we smoothed the timeseries of x/y coordinates at each original timepoint with a Kalman smoother using a dynamic linear model. Fourth, we linearly interpolated the locations to a 10 s resolution based on ODBA, where we considered the animal to be stationary between multiple timepoints if the accelerometer signal suggested the animal was not moving. Fifth, we fitted an X-spline through the data, where we gave the linearly ODBA-interpolated locations a smaller weight, and sampled the fitted spline on a regular 1-min resolution. These pre-processing steps resulted in the modelled animal trajectory data, composed of spatial locations every minute, and averaged ODBA statistics per step (i.e., the segments between consecutive coordinates). These data were used as input for the next steps in the analyses. In contrast to the animal data, the raw intrusion data were of a high temporal resolution and spatial accuracy so that we only needed to subset the data in order to acquire 1-min resolution time-synchronized intrusion trajectories.

The first three parts of the data pre-processing were only needed because of firmware issues in our custom-made sensors. Without these issues, a simple denoising technique like a Kalman filter will suffice.

### Feature engineering and processing

We computed a plethora of human-engineered features from the animal trajectories, ODBA data, weather data and several GIS layers with environmental data from the study area (summarized in Supplementary Table S1). All features were computed such that they could not directly be linked to specific points in space or time (by computing movement features relative to the environmental variables), so that only behavioral patterns and abnormalities therein could be linked to intrusion presence. After engineering these base features, we transformed certain features (after visual inspection of the histograms) to approximately symmetric distributions using logarithms. Then we truncated the distributions to the lower and upper 0.001 percentile to correct possible outliers. After that, we standardized all computed features to zero mean and unit variance per species. We also computed scaled versions of selected features by subtracting the mean and dividing by the variance of the selected features per reference set to capture deviations from normal behavior: (1) per area (characterized by a 30 by 30 m neighborhood around each grid cell), (2) per time of day (morning, midday, afternoon) in a period of 5 weeks around each intrusion or control, (3) per area per time of day per 5 weeks, and (4) per individual sentinel per time of day per 5 weeks (Supplementary Table S1). Furthermore, after computing and standardizing the features, we computed more features by applying moving window computations (5 min centered, 10 and 20 min lagging, and the difference between these: 5 min centered minus 10 and 20 min lagging) on the standardized features to capture (the change in) the recent history of animal movement descriptors (mean and standard deviation of all features, fitted Mean Squared Displacement exponential function parameters, net-gross distance ratio and variance of log First Passage Times). Finally, we discretized all features to ordinal values to avoid odd-, fat- and heavy-tailed distributions. In total we computed 2117 features describing different aspects of movement geometry of individual trajectories, herd topology and the interactions with landscape variation.

### Subsetting and dimensionality reduction

Before analyzing the computed animal movement features, we applied some filtering on the data. We removed all periods with an experimental intrusion during which there were less than 30 active animal sensors in total. We also removed data of both animals and intrusion when they were close to the reserve’s main gate in order to avoid dilution of the data with other known disturbances. This resulted in 57 intrusions that were selected for further analyses. For every intrusion we selected control data of the same period one or 2 days earlier or later during which no intrusion took place, resulting in an approximately balanced intrusion-control dataset. Furthermore, we removed data from animals that were located within 250 m and within 20 min of a vehicle moving through the area that was not part of our experiment.

For each feature, we computed 4 importance metrics based on binary labelled data: records associated to locations within 1 km from the intrusion (subscript 1) versus an equally-sized random selection of data points during control periods (subscript 0): Mahalanobis distance, marginality (computed as $\frac{{\mu }_{1}-{\mu }_{0}}{{\sigma }_{0}}$, for sample mean $\mu$ and sample standard deviation $\sigma$), specialization (computed as $\frac{{\sigma }_{1}}{{\sigma }_{0}}$) and the Mean Decrease Accuracy of a Random Forest classifier (with default hyperparameters). We then ranked the features according to their importance and selected a feature for further analyses if it occurred in the top 125 features for any of the 4 importance measures described above (resulting in a total of 361 selected features). Subsequently, we converted the selected features per main feature class (Supplementary Table S1) to principal components, keeping those principal components that capture the most variation (in total 95%), which resulted in 99 selected components in total. Finally, we transformed these components again via a second principal component analysis, now across all the selected 99 components. In subsequent training of the animal behavior classifier, we optimized the total number of included components as a hyperparameter, which resulted in the first 8 principal components in the best performing classifier.

### Labelling

We labelled the sentinel movement data through visual inspection of the animal and intruder trajectories, where we considered the animals’ behavior to be undisturbed when the animal was not near an intrusion, or when the animal was close to an intrusion yet did not visually display a change in behavior. However, when the animal was near the intrusion and displayed a sudden or gradual behavioral change in response to intrusion proximity, we labelled the data as ‘flight’ (changing the movement direction away from the intrusion, possibly with increased speed) or ‘regroup’ (when individuals clustered together). In total, only ca. 1% of the animal data were associated to either flight or regroup behavior (which we will refer to as ‘response’ behavior). A few animals also appeared to exhibit behavior we could label as ‘freeze’, i.e., halting movement in the proximity of the intrusion, yet this class was too underrepresented to be accurately predicted and hence dropped from the final dataset. Furthermore, we assigned a qualitative measure of intensity to each labelled behavioral response (‘low’, ‘medium’, ‘high’) to describe how visually pronounced this response was. Besides the supervised labelling based on visual inspection of behavioral responses via video animations of the trajectories, we also labelled data using an unsupervised *k*-means nearest neighbor classifier, where we clustered the feature space consisting of the 99 features selected as described above into 25 clusters per species.

### Animal behavior classification

We trained an RBF kernel C-classification Support Vector Machine (SVM) with a subsequent moving window over the outputted probabilities to distinguish undisturbed versus response behavior. In the training datasets we only included the data separated by more than 1 km from the intrusion and labelled as ‘undisturbed’, and removed 90% thereof to train algorithms with a more balanced dataset. Furthermore, we only trained and validated on data with intrusions present in the area. We trained another SVM to distinguish the flight response from the regroup response. All computations were done in R 3.5.0 with the e1071 package on the Linux High Performance Cluster of Wageningen University and Research. We optimized the following hyperparameters and model settings during the training phase for the Average Precision via a grid search (with the selected values between brackets):

- gamma (undisturbed-response: 10^–3.2^; flight-regroup: 10^–2.0^);
- cost (undisturbed-response: 10^–2.2^; flight-regroup: 10^–1.5^);
- number of principal components to include as features (undisturbed-response: 8; flight-regroup: 12);
- species-specific models versus one model with species dummy variables included in the features (species-specific models);
- specific models for the different times of day versus one model with time of day dummy variables (one model);
- response intensities to include in the training data (only medium and high intensities);
- weights to assign to the classes (equal weights);
- the quantile to be computed of the SVM probabilities by the moving window (100%, i.e., maximum value);
- the alignment of the moving window (centered);
- the size of the moving window (15 min on both sides).

The best model was selected via a leave-one-intrusion-out cross-validation approach. We summarized the predictive performance by computing the Average Precision of the least occurring class (i.e., ‘response’ for the undisturbed-response model: 46%, Supplementary Fig. S2; and ‘regroup’ for the flight-regroup model: 80%, Supplementary Fig. S3). After having computed these probabilities with an SVM and a temporal window smoother, we tried to improve the predicted performance by including the predicted animal response probabilities of nearby animals. However, this spatial explicit approach hardly improved the predictive performance, indicating that the spatial contextualization of behavioral response was sufficiently captured by the computed features. We therefore did not include this spatial contagion effect of predicted animal response probabilities in the final analysis.

### System classification—detection

Based on the predicted SVM response probabilities and feature cluster analysis, we computed summary features per 15 min of each intrusion and control period. These summary features related to the odds ratios of the probability of association of unsupervised clusters with intrusions versus controls, the SVM predicted probabilities of behavioral response, and several features describing the values (and its spatial structure, e.g., clustering or autocorrelation) of these SVM predicted response probabilities. After computing summary features per 15 min, we summarized them even further for the intrusions versus controls using the following eight statistics: mean, standard deviation, minimum, maximum, mean of the lagged differences, standard deviation of the lagged differences, minimum of the lagged differences and maximum of the lagged differences.

After computing the summary features, we build a logistic regression classifier to distinguish intrusions from controls. To create a parsimonious model, we iteratively added features to the model and evaluated its performance after each iteration. We evaluated the performance based on the model accuracy and performed validation through 25 times twofold cross-validation in a stratified way (by 25 times choosing a balanced random sample of intrusions and controls). We determined the sequence of adding features to the model by performing an independent two-sample t-test for each feature between the intrusions and controls. The feature with the largest t-value was then added to the model. After each feature addition, we removed its correlation with the remaining features using linear regressions with the added feature as independent variable and the remaining features as dependent variables, from which we extracted the residuals, standardized them to zero mean and unit variance, and applied the t-tests again. The (original) feature with the largest t-value was then added to the model again. This procedure was repeated until all features were ordered corresponding to their “importance”. We then performed logistic regressions without interactions between the features for an increasing number of features (Supplementary Fig. S4). The model already performed quite accurately with only 7 features (86.1% accuracy ± SD 3.3%, precision 82.6% ± SD 6.9%, recall 89.2% ± SD 5.1%). However, with 20 features and 2-way interactions the model achieved the maximum accuracy (90.9%).

### System classification—localization

The data gathered during intrusions that were correctly predicted as such by the detection classifier were used to train the intrusion localization algorithm. The probability surface of the location of the intrusion was fitted relative to that of the sentinel animals using:

$${O}_{i,j} \sim \frac{{p}_{j}\left({f}_{wn}\left({\theta }_{i,j},{\mu }_{j},{\rho }_{1}\right) {f}_{ln}\left({\gamma }_{i,j},{\mu }_{1},{\sigma }_{1}\right)\right) \left(1-{p}_{j}\right) \left({f}_{wn}\left({\theta }_{i,j},{\mu }_{j},{\sigma }_{0}\right) {f}_{ln}\left({\gamma }_{i,j},{\mu }_{0},{\sigma }_{0}\right)\right)}{{f}_{wn}\left({\theta }_{i,j},{\mu }_{j},{\rho }_{0}\right) {f}_{ln}({\gamma }_{i,j},{\mu }_{0},{\sigma }_{0})}$$

where ${O}_{i,j}$ is the odds ratio of intrusion presence at location $i$ evaluated for individual $j$, ${p}_{j}$ is the SVM-predicted probability that individual $j$ is exhibiting response behavior. The function ${f}_{wn}$ is the wrapped normal probability density function, ${\theta }_{i,j}$ is the direction from location $i$ to the location of the focal animal $j$, ${\mu }_{j}$ is the movement direction of individual $j$, ${\rho }_{1}$ and ${\rho }_{0}$ are the standard deviations of the unwrapped distributions. The function ${f}_{ln}$ is the lognormal probability density function, where ${\gamma }_{i,j}$ is the distance of location $i$ to $j$, ${\mu }_{1}$ and ${\mu }_{0}$ as well as ${\sigma }_{1}$ and ${\sigma }_{0}$ are the log-normal distribution parameters (respectively log-mean and log-sd).

The parameters ${\mu }_{1}$, ${\sigma }_{1}$ and ${\rho }_{1}$ capture the geometry of intrusion-animal topology for animals that exhibited a predicted behavioral response to the intrusion. Similarly, ${\mu }_{0}$, ${\sigma }_{0}$ and ${\rho }_{0}$ are the corresponding parameters for animals that were predicted to be undisturbed. The parameters ${\mu }_{1}$, $\mathrm{log}({\sigma }_{1})$ and $\mathrm{log}({\rho }_{1})$ were fitted to the data assuming a 3rd order polynomial relationship to ${t}_{s}$: the time (in minutes) since the start of the predicted behavioral response (using the maximum *F*_*1*_ classification score). Since the behavioral response signature is lost over time, we truncated ${t}_{s}$ to 45 min (thus ${t}_{s}>45$ min was set to ${t}_{s}=45$). The parameters ${\mu }_{0}$, ${\sigma }_{0}$ and ${\rho }_{0}$ were estimated using the data of the controls and with randomly generated intrusion locations in the study area, in order to correct for the effects of geometry of the study area on the predicted response surfaces. The probability surface ${P}_{i}$ was then calculated as:

$${P}_{i}=\alpha \sum_{j}{O}_{i,j}$$

where $\alpha$ is a normalization constant so that ${P}_{i}$ integrates to 1 over the area covered by the rectangular axis-aligned bounding box around the study area.

To measure the prediction accuracy of each localization surface, we simplified each surface to a point coordinate located at the location of maximum probability, and computed the Euclidian distance to the known true position of the intrusion. We then summarized each experimental intrusion by selecting the 10 prediction surfaces with the most condense highest probability density, i.e., those in which the top 5% probability density is contained in the smallest, most condense, area. The spatial error of the localization prediction associated with these selected predictions was further summarized by taking the average Euclidian distance over the 10 selected predictions.

## Supplementary Information

Supplementary Information 1.

Supplementary Information 2.

Supplementary Information 3.

## Data Availability

Our data and code are available in the 4TU.ResearchData repository: 10.4121/13900106^50^.

## References

[1] Scheffers BR, Oliveira BF, Lamb I, Edwards DP (2019). Global wildlife trade across the tree of life. Science (80-.)..

[2] Felbab-Brown V (2017). The Extinction Market.

[3] Chen F (2016). Poachers and snobs: demand for rarity and the effects of antipoaching policies. Conserv. Lett..

[4] Ceballos G, Ehrlich PR, Dirzo R (2017). Biological annihilation via the ongoing sixth mass extinction signaled by vertebrate population losses and declines. Proc. Natl. Acad. Sci..

[5] Veríssimo D, Wan AKY (2019). Characterizing efforts to reduce consumer demand for wildlife products. Conserv. Biol..

[6] Duffy R (2014). Waging a war to save biodiversity: the rise of militarized conservation. Int. Aff..

[7] O’Donoghue P, Rutz C (2016). Real-time anti-poaching tags could help prevent imminent species extinctions. J. Appl. Ecol..

[8] Cooper WE, Blumstein DT (2015). Escaping From Predators: An Integrative View of Escape Decisions. Escaping From Predators.

[9] Zbyryt A (2018). Do wild ungulates experience higher stress with humans than with large carnivores?. Behav. Ecol..

[10] Frid A, Dill LM (2002). Human-caused disturbance stimuli as a form of predation risk. Conserv. Ecol..

[11] Katzner TE, Arlettaz R (2020). Evaluating contributions of recent tracking-based animal movement ecology to conservation management. Front. Ecol. Evol..

[12] Williams HJ (2020). Optimizing the use of biologgers for movement ecology research. J. Anim. Ecol..

[13] Stankowich T (2008). Ungulate flight responses to human disturbance: a review and meta-analysis. Biol. Conserv..

[14] Gallagher AJ, Creel S, Wilson RP, Cooke SJ (2017). Energy landscapes and the landscape of fear. Trends Ecol. Evol..

[15] Ihwagi FW (2018). Night-day speed ratio of elephants as indicator of poaching levels. Ecol. Indic..

[16] Halsey LG (2016). Terrestrial movement energetics: current knowledge and its application to the optimising animal. J. Exp. Biol..

[17] Wilson RP, Quintana F, Hobson VJ (2012). Construction of energy landscapes can clarify the movement and distribution of foraging animals. Proc. R. Soc. B Biol. Sci..

[18] Gaynor KM, Brown JS, Middleton AD, Power ME, Brashares JS (2019). Landscapes of fear: spatial patterns of risk perception and response. Trends Ecol. Evol..

[19] Westley PAH, Berdahl AM, Torney CJ, Biro D (2018). Collective movement in ecology: from emerging technologies to conservation and management. Philos. Trans. R. Soc. B Biol. Sci..

[20] Calabrese JM (2018). Disentangling social interactions and environmental drivers in multi-individual wildlife tracking data. Philos. Trans. R. Soc. B Biol. Sci..

[21] Hamilton WD (1971). Geometry for the selfish herd. J. Theor. Biol..

[22] Evans DA, Stempel AV, Vale R, Branco T (2019). Cognitive control of escape behaviour. Trends Cogn. Sci..

[23] Bode NWF, Faria JJ, Franks DW, Krause J, Wood AJ (2010). How perceived threat increases synchronization in collectively moving animal groups. Proc. R. Soc. B Biol. Sci..

[24] Ioannou CC, Ramnarine IW, Torney CJ (2017). High-predation habitats affect the social dynamics of collective exploration in a shoaling fish. Sci. Adv..

[25] Berdahl A, Torney CJ, Ioannou CC, Faria JJ, Couzin ID (2013). Emergent Sensing of complex environments by mobile animal groups. Science (80–.)..

[26] Lima SL (1995). Back to the basics of anti-predatory vigilance: the group-size effect. Anim. Behav..

[27] Charuvi A (2020). A physiological cost to behavioural tolerance. Behav. Process..

[28] Darimont CT, Fox CH, Bryan HM, Reimchen TE (2015). The unique ecology of human predators. Science (80–.)..

[29] Suraci JP, Clinchy M, Zanette LY, Wilmers CC (2019). Fear of humans as apex predators has landscape-scale impacts from mountain lions to mice. Ecol. Lett..

[30] Zanette LY, Clinchy M (2020). Ecology and neurobiology of fear in free-living wildlife. Annu. Rev. Ecol. Evol. Syst..

[31] Miller JRB, Ament JM, Schmitz OJ (2014). Fear on the move: predator hunting mode predicts variation in prey mortality and plasticity in prey spatial response. J. Anim. Ecol..

[32] Thaker M (2011). Minimizing predation risk in a landscape of multiple predators: effects on the spatial distribution of African ungulates. Ecology.

[33] Creel S (2018). The control of risk hypothesis: reactive vs. proactive antipredator responses and stress-mediated vs. food-mediated costs of response. Ecol. Lett..

[34] Ciuti S (2012). Effects of humans on behaviour of wildlife exceed those of natural predators in a landscape of fear. PLoS ONE.

[35] Proffitt KM, Grigg JL, Hamlin KL, Garrott RA (2009). Contrasting effects of wolves and human hunters on Elk behavioral responses to predation risk. J. Wildl. Manag..

[36] Montgomery RA, Macdonald DW, Hayward MW (2020). The inducible defences of large mammals to human lethality. Funct. Ecol..

[37] Goumas, M., Lee, V. E., Boogert, N. J., Kelley, L. A. & Thornton, A. The Role of animal cognition in human–wildlife interactions. *Front. Psychol.* **11**, 589978 (2020). 10.3389/fpsyg.2020.589978 PMC7672032 33250826

[38] Reif JS (2011). Animal sentinels for environmental and public health. Public Health Rep..

[39] Rabinowitz P, Wiley J, Odofin L, Wilcox M, Dein FJ (2008). Animals as sentinels of chemical terrorism agents: an evidence-based review. Clin. Toxicol..

[40] Wikelski, M. *et al.* Potential short-term earthquake forecasting by farm-animal monitoring. *bioRxiv* (2020). 10.1101/2020.01.19.911313.

[41] Woith H, Petersen GM, Hainzl S, Dahm T (2018). Review: can animals predict earthquakes?. Bull. Seismol. Soc. Am..

[42] Catala A (2019). Dogs demonstrate the existence of an epileptic seizure odour in humans. Sci. Rep..

[43] Bakeman U (2019). Detection of impending aggressive outbursts in patients with psychiatric disorders: violence clues from dogs. Sci. Rep..

[44] Wikelski M, Tertitski G (2016). Ecology: living sentinels for climate change effects. Science (80–.).

[45] Weimerskirch H (2020). Ocean sentinel albatrosses locate illegal vessels and provide the first estimate of the extent of nondeclared fishing. Proc. Natl. Acad. Sci..

[46] Mehran, R., Oyama, A. & Shah, M. Abnormal crowd behavior detection using social force model. in *2009 IEEE Conference on Computer Vision and Pattern Recognition* 935–942 (IEEE, 2009). 10.1109/CVPR.2009.5206641.

[47] Gleiss AC, Wilson RP, Shepard ELC (2011). Making overall dynamic body acceleration work: On the theory of acceleration as a proxy for energy expenditure. Methods Ecol. Evol..

[48] CyberTracker. CyberTracker. https://www.cybertracker.org/. Accessed: 10 May 2017.

[49] R Core Team (2020). R: A Language and Environment for Statistical Computing.

[50] Eikelboom J A J, de Knegt H J (2021). 4TU.ResearchData.

