# Supplementary material for: Timely poacher detection and localization using sentinel animal movement
Source: Sci Rep. 2021 Feb 25;11:4596. doi: 10.1038/s41598-021-83800-1 (PMC7907380; doi:10.1038/s41598-021-83800-1)
Supplement: Supplementary file 1 — Supplementary Information 1. [file 41598_2021_83800_MOESM1_ESM.pdf]

Timely poacher detection and localization using sentinel animal movement

Henrik J. de Knegt[1‡*], Jasper A.J. Eikelboom[1‡*], Frank van Langevelde[1,2], W. François Spruyt[3], Herbert H.T. Prins[1,4]

[1]Wildlife Ecology and Conservation Group, Wageningen University and Research, Droevendaalsesteeg 3a, 6708 PB Wageningen, Netherlands.

[2]School of Life Sciences, Westville Campus, University of KwaZulu-Natal, Durban 4000, South Africa.

[3]Welgevonden Game Reserve, P.O. Box 433, Vaalwater, South Africa.

[4]Department of Animal Sciences, Wageningen University and Research, De Elst 1, 6708 WD Wageningen, Netherlands.

*Correspondence to: Jasper A.J. Eikelboom (jasper.eikelboom@wur.nl) or Henrik J. de Knegt (henjo.deknegt@wur.nl).

‡These authors contributed equally to this work.

Henrik J. de Knegt: 0000-0003-2105-6970

Jasper A.J. Eikelboom: 0000-0002-5107-4427

Frank van Langevelde: 0000-0001-8870-0797

Herbert H.T. Prins: 0000-0003-1131-5107

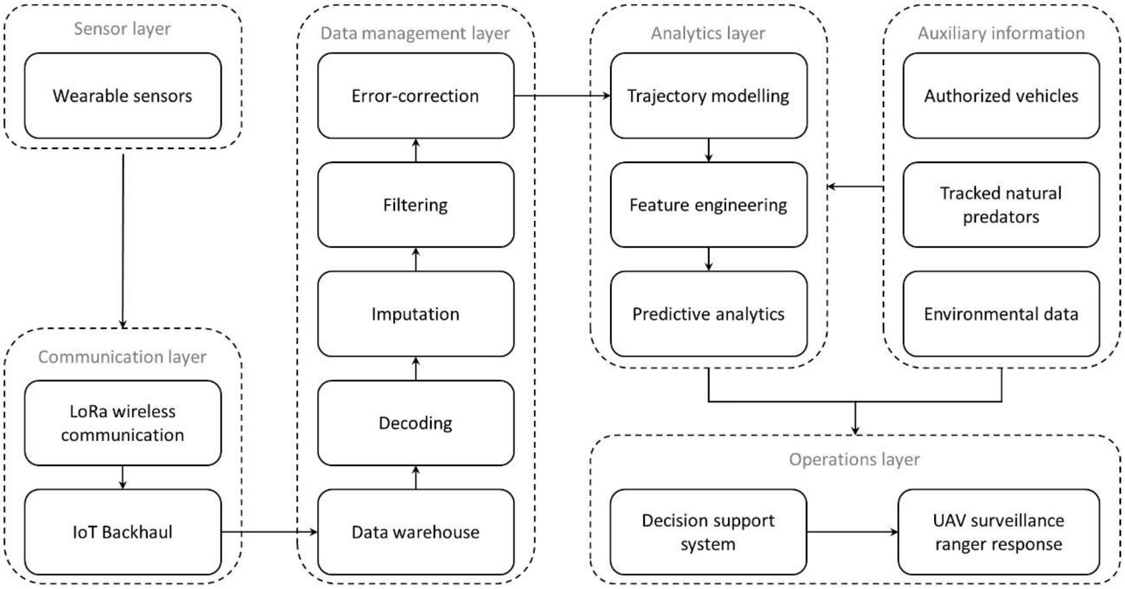

Fig S1. Schematic diagram of our proposed sentinel-based poacher early warning system.

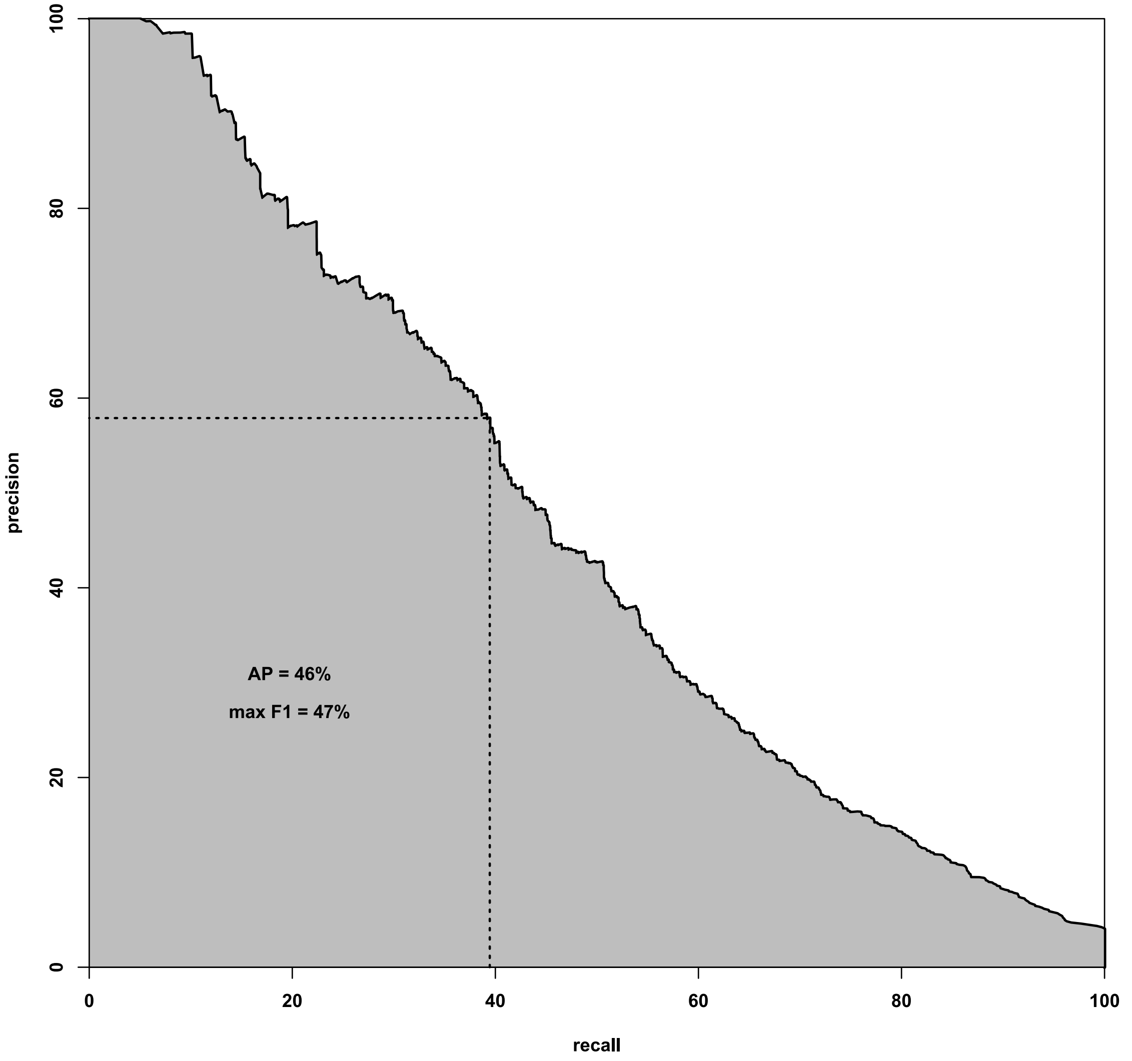

Fig. S2. Precision-recall curve for 'response' of the undisturbed-response model. The dotted line indicates the maximum harmonic mean of precision and recall (F1-score).

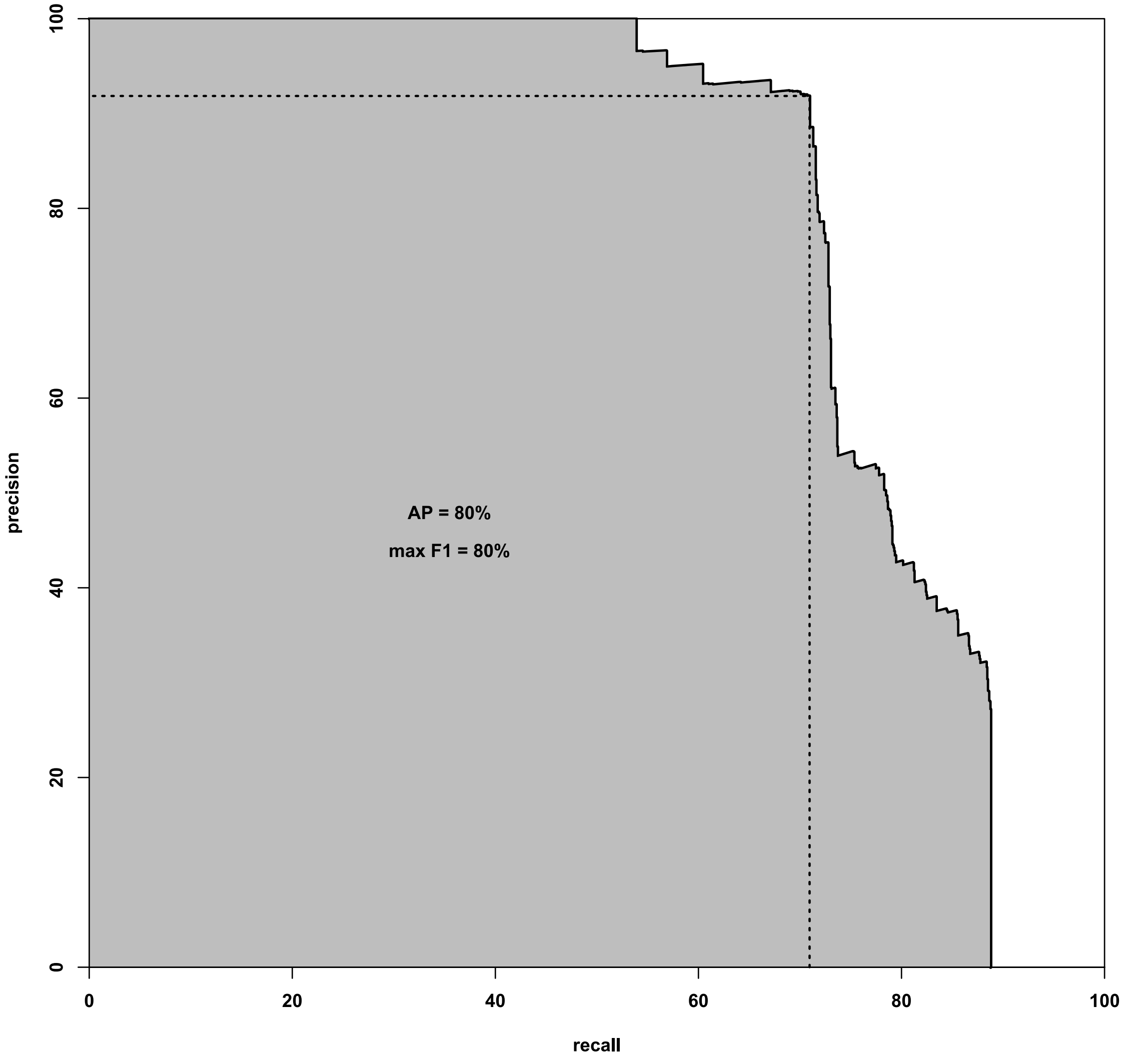

Fig. S3. Precision-recall curve for ‘regroup’ of the flight-regroup model. The dotted line indicates the maximum harmonic mean of precision and recall (F1-score).

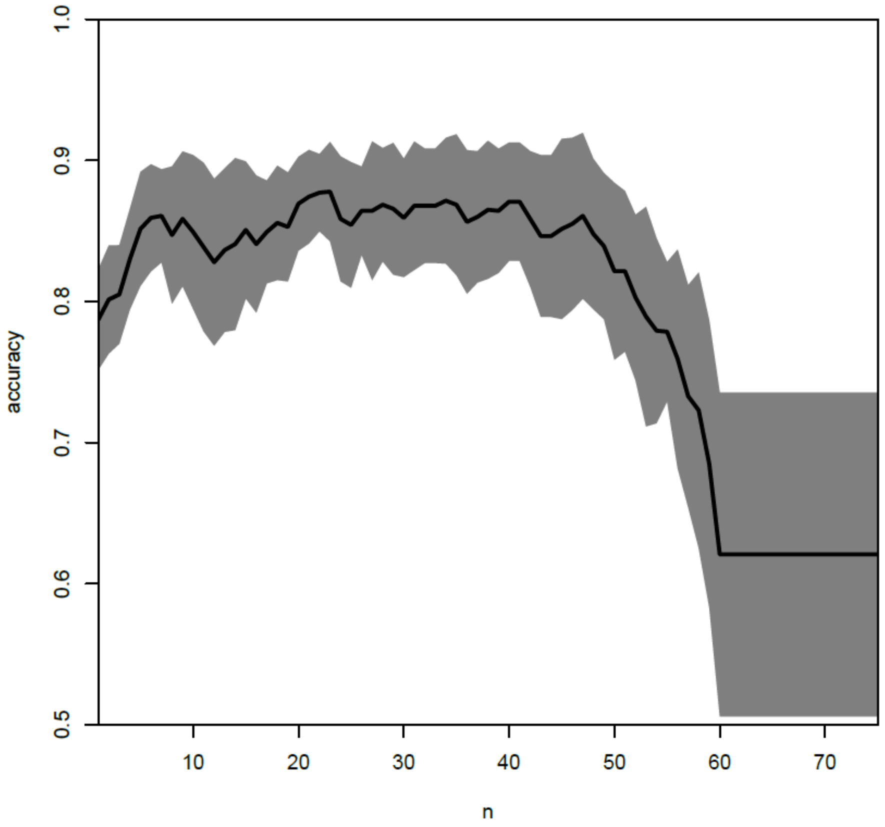

Fig. S4. Accuracy vs. the number of features of the intrusion classification logistic regressions.

Table S1. Computed features per record. To capture deviations from normal behavior, features were standardized 0) per species and selected features were also standardized 1) per area (a 30 x 30 m neighborhood centered on each grid cell), 2) per time of day (morning, midday, afternoon) in the five weeks around each experimental intrusion or control, 3) per area per time of day per five weeks, and 4) per individual per time of day per five weeks. The means and variances used for standardization were computed from data in the absence of experimental intrusions. In total, this resulted in 2117 computed features (the set below, plus species identification and time of day; morning, midday or afternoon).

Feature main class	Feature sub class	Feature	Standardization
Individual movement geometry	Speed	Speed	0,2,3,4
		Scale parameter of a fitted SSM for speed[1]	0,1,3
		Shape parameter of a fitted SSM for speed[1]	0,1,3
		Value of the cumulative distribution function given a fitted SSM for speed[1]	0,1,3
		Log-likelihood of a fitted SSM for speed[1]	0
		Scaled deviation from maximum value of the pdf of a fitted SSM for speed[1] (with negative values when speed < mode)	0
	Relative headings	Absolute turning angle (TA)	0,2,3,4
		Cosine TA	0,2,3,4
		Concentration parameter of a fitted SSM for TA[2]	0,1,3
		Log-likelihood of a fitted SSM for TA[2]	0
		Scaled deviation from maximum value of the pdf of a fitted SSM for TA[2]	0
		Absolute centripetal turning angle	0,2,3,4
		Cosine centripetal turning angle	0,2,3,4
		Cosine relative angle to terrain aspect	0
		Cosine relative angle to feeding site	0
		Cosine relative angle to average movement direction	0,1,3
		Cosine relative angle to nearest track	0
		Cosine relative angle to nearest road	0
		Cosine relative angle to wind direction	0
		Cosine relative angle to terrain aspect, weighted for slope	0
		Cosine relative angle to feeding site, weighted for distance to feeding site	0

		Cosine relative angle to average movement direction, weighted for rho of movement directions	0,1,3
		Cosine relative angle to nearest track, weighted for distance to track	0
		Cosine relative angle to nearest road, weighted for distance to road	0
		Cosine relative angle to wind direction, weighted for wind speed	0
	Acceleration	Acceleration	0,2,3,4
		Angular acceleration	0,2,3,4
		Centripetal acceleration	0,2,3,4
		Centripetal angular acceleration	0,2,3,4
		Tangential acceleration	0,2,3,4
	Velocity	Absolute tangential velocity	0,2,3,4
		Centripetal velocity	0,2,3,4
	Compound features	First Passage Time (FPT) at 3m radius	0,2,3,4
		FPT at 9m radius	0,2,3,4
		FPT at 27m radius	0,2,3,4
		Slope of the radius vs FPT relationship	0,2,3,4
		Rate of exponential decay of the Velocity Autocorrelation Function (VAF) up to 15-minute time lag	0,2,3,4
		Rate of exponential decay of the VAF up to 30-minute time lag	0,2,3,4
Accelerometer-based features	Fraction moved	Estimated fraction of time moving	0,1,2,3,4
	Mean	Mean mean acceleration	0,2,3,4
		Mean standard deviation acceleration	0,2,3,4
		Mean peak acceleration	0,2,3,4
	Standard deviation	Standard deviation average acceleration	0,2,3,4
		Standard deviation standard deviation acceleration	0,2,3,4
		Standard deviation peak acceleration	0,2,3,4
Collective movement features (both computed across species, as well as for conspecifics only)	Relative angles	Cosine of relative heading compared to the weighted mean of headings of surrounding animals[3]	0,2,3,4
		Length of the weighted resultant vector of headings of surrounding animals[3]	0,2,3,4
		Cosine of relative heading compared to the weighted mean of headings of surrounding animals[3], multiplied by the weighted resultant vector length	0

		Cosine of relative heading compared to the weighted mean of headings of surrounding animals, multiplied by the weighted resultant vector length and sum of weights[3]	0
		Cosine of the relative heading towards the herd-center (the distance weighted mean coordinate of nearby animals[3])	0,2,3,4
	Distance	Distance towards the herd-center	0,2,3,4
		Distance towards the nearest animal	0,2,3,4
		Sum of distance-based weights[3]	0,2,3,4
Indices of space usage (at both the individual and species level)	Index of Habitat Suitability[4] (HSI)	HSI value at location of next record ('used')	0
		Mean and sd of HSI value in neighboring grid cells	0
		Measure of step selection: (used - mean) / sd	0
	Utilization distribution via Kernel Density Estimation[5] (KDE)	KDE value at location of next record ('used')	0
		Mean and sd of KDE value in neighboring grid cells	0
		Measure of step selection: (used - mean) / sd	0

[1] Fitted state-space model (SSM) for speed using a lognormal distribution, where the scale parameter and the logarithm of the shape parameter are related to environmental predictors via a linear predictor.
[2] Fitted SSM for turning angle using a wrapped Cauchy distribution where the expectation is 0 (i.e. no change of direction) and the concentration parameter (via logit link) related to environmental predictors via a linear predictor.
[3] The weight of surrounding animals decays with distance from the focal individual proportional to a normal distribution with mean 0 and standard deviation of 100m (scaled to have weight 1 at distance 0).
[4] MaxEnt habitat suitability prediction based on environmental predictors fitted per species per time of day in a window of 5 weeks around an experiment/control.
[5] Using an isotropic bivariate gaussian kernel with a bandwidth of 10m.

Movie S1 (separate file). Labelled animation of one experimental intrusion, coupled with visualizations of all three layers of the analyses: behavioral response classification, intrusion detection, and intrusion localization.
