# Supplementary material for: Timely poacher detection and localization using sentinel animal movement
Source: Sci Rep. 2021 Feb 25;11:4596. doi: 10.1038/s41598-021-83800-1 (PMC7907380; doi:10.1038/s41598-021-83800-1)
Supplement: Supplementary file 3 — Supplementary Information 3. [file 41598_2021_83800_MOESM3_ESM.pdf]

Thursday, 23 April 2020

To whom it may concern:

Welgevonden Game Reserve (WGR) is a private entity under South African law registered as a non-for-profit company [company number 1993/004923/08 (CPIC documents attached)]. Its aim is to conserve and preserve wildlife on its properties. WGR operates within the ethical guidelines advised by the South African Ministry of the Environment. The operations of the Reserve are carried out under the National Environmental Management, Protected Areas Act (NEM: PAA) (Act No. 57 of 2003) and are also subject to the provincial Limpopo Environmental Management Act 7 of 2003.

According to the South African laws, the work described in the manuscript is the outcome of procedures aimed at optimizing the management of WGR (~38,000 ha), namely, looking after the natural values of the reserve, maintaining healthy populations of protected species in the reserve, and the protection of its endangered rhino population. The participation of Wageningen University (the Netherlands) has been sought solely for idea development, planning and data analyses aimed at increasing the chances of protecting WGR's rhino population.

For the work described in this manuscript, the Board and CEO of WGR decided that it was necessary to deploy "sentinel animals" as described, for which animals from outside the working area had to be introduced into that area. The board and CEO of WGR regarded the deployment of these sentinel animals solely as a management action. Catching, transport, release, (GPS-)collaring, temporary holding, etc., of a species is carried out under the P3 Exemption Permit and Threatened and Protected Species [TOPS] Permit), which is issued to a property that can show reasonable control over the movements of that species and allows the permit holder to manage the game species listed on that permit. WGR holds these permits, and only purchases animals in a legal fashion from similar other properties with such permits.

Collaring and sedation of animals followed procedures prescribed by South African law which entails that this can be done by qualified and nationally registered veterinarians only. Neither staff nor students of Wageningen University (or any other research organization) have been allowed to handle any animal before, during or after the work reported in this manuscript.

After the Board and CEO decided to carry out the introductions of potential sentinel animals, security personnel of the Reserve executed the "intrusion experiments" during their regular tasks on terrain of the Reserve and were informed that they (as usual for their task) walked or drove in the terrain with large mammals that can possibly be harmful and lethal. However, the "intrusion experiment" was carried out in the so-called Breeding Camp only, which is a 1200 ha-fenced block of the Reserve without elephants or lions. This security personnel followed all safety courses, drills and practices that are prescribed by national law and regulation, were and are authorised to carry guns, and according their instructions carried GPS-receivers and radiocommunication equipment for their safety. All were fully informed about the "intrusion experiments", its purpose and their tasks, and gave their informed consent. The Head of the anti-poaching division of the Reserve was fully cognisant of the experiment and vouchsafed that it fell completely within the normal modus operandi of the staff of his division.

WELGEVONDEN GAME RESERVE NPC
REG. NO. 1993/004923/08 VAT REG. NO. 4100139692
www.welgevonden.org
info@welgevonden.org

P.O. BOX 433, VAALWATER, 0530, SOUTH AFRICA. TEL: +27 87 813 0501, FAX: +27 86 501 8400

DIRECTORS: P DE SMIDT D DRENNAN JI IBBOTSON HJ KRIEK JH LAMBERT SD MCCARTNEY (CHIEF EXECUTIVE)
VZ MNTAMBO JL SEELIGER DJ SMULLEN WF SPRUYT (CHAIRMAN) FT VOGEL

COMPANY SECRETARY: NETTIE MOFFAT (FLUIDROCK GOVERNANCE [PTY] LTD)

The Board of the Welgevonden Game Reserve is the relevant authority for any activity carried out in the Reserve. The Board and the CEO gave their permission for the GPS-collaring of the sentinel animals and the "intrusion experiments" as described in this manuscript. It is the intention of the Board to deploy the sentinel animal concept as described in this manuscript to the whole of the Reserve to investigate its utility for the management of the whole of the Reserve but also to make this knowledge available to other protected areas.

Signed Dated 23 April 2020

Chief Executive Officer

Welgevonden Game Reserve
